# Prefrontal cortical and striatal transcriptional responses to the reinforcing effect of repeated methylphenidate treatment in the spontaneously hypertensive rat, animal model of attention-deficit/hyperactivity disorder (ADHD)

**DOI:** 10.1186/1744-9081-10-17

**Published:** 2014-05-06

**Authors:** Ike dela Peña, Hee Jin Kim, Aeree Sohn, Bung-Nyun Kim, Doug Hyun Han, Jong Hoon Ryu, Chan Young Shin, Minsoo Noh, Jae Hoon Cheong

**Affiliations:** 1 Uimyung Research Institute for Neuroscience, Sahmyook University, 26-21 Kongreung-2-dong, Hwarangro- 815 Nowon-gu, Seoul 139-742, Korea; 2 Division of Child and Adolescent Psychiatry, Clinical Research Institute, Seoul National University Hospital, 28 Yungundong, Chongrogu, Seoul 110-744, Korea; 3 Department of Psychiatry, Chung-Ang University Medical School, 102 Heukseok-ro, Dongjak-gu, Seoul 156-755, Korea; 4 Department of Oriental Pharmaceutical Science, Kyung Hee East–west Pharmaceutical Research Institute, College of Pharmacy, Kyung Hee University, Seoul 472-864, Korea; 5 Department of Neuroscience, School of Medicine, Konkuk University, Seoul 143-701, Korea; 6 Natural Products Research Institute, College of Pharmacy, Seoul National University, 1 Gwanak-ro, Gwanak-gu, Seoul 151-742, Korea

**Keywords:** Methylphenidate, ADHD, Gene expression, Addiction

## Abstract

**Background:**

Methylphenidate is the most commonly used stimulant drug for the treatment of attention-deficit/hyperactivity disorder (ADHD). Research has found that methylphenidate is a “reinforcer” and that individuals with ADHD also abuse this medication. Nevertheless, the molecular consequences of long-term recreational methylphenidate use or abuse in individuals with ADHD are not yet fully known.

**Methods:**

Spontaneously hypertensive rats (SHR), the most validated and widely used ADHD animal model, were pretreated with methylphenidate (5 mg/kg, i.p.) during their adolescence (post-natal day [PND] 42–48) and tested for subsequent methylphenidate-induced conditioned place preference (CPP) and self-administration. Thereafter, the differentially expressed genes in the prefrontal cortex (PFC) and striatum of representative methylphenidate-treated SHRs, which showed CPP to and self-administration of methylphenidate, were analyzed.

**Results:**

Genome-wide transcriptome profiling analyses revealed 30 differentially expressed genes in the PFC, which include transcripts involved in apoptosis (e.g*. S100a9, Angptl4, Nfkbia*), transcription (*Cebpb, Per3*), and neuronal plasticity (*Homer1, Jam2, Asap1*). In contrast, 306 genes were differentially expressed in the striatum and among them, 252 were downregulated. The main functional categories overrepresented among the downregulated genes include those involved in cell adhesion (e.g. *Pcdh10, Ctbbd1, Itgb6*), positive regulation of apoptosis (*Perp, Taf1, Api5*), (*Notch3, Nsbp1, Sik1*), mitochondrion organization (*Prps18c, Letm1, Uqcrc2*), and ubiquitin-mediated proteolysis (*Nedd4, Usp27x, Ube2d2*).

**Conclusion:**

Together, these changes indicate methylphenidate-induced neurotoxicity, altered synaptic and neuronal plasticity, energy metabolism and ubiquitin-dependent protein degradation in the brains of methylphenidate-treated SHRs, which showed methylphenidate CPP and self-administration. In addition, these findings may also reflect cognitive impairment associated with chronic methylphenidate use as demonstrated in preclinical studies. Future studies are warranted to determine the clinical significance of the present findings with regard to long-term recreational methylphenidate use or abuse in individuals with ADHD.

## Background

Central nervous system (CNS) stimulants (e.g. methylphenidate and amphetamines) are recommended as first line medications for attention-deficit/hyperactivity disorder (ADHD [1]. Among the psychostimulants, methylphenidate is the most commonly prescribed [1]. Methylphenidate blocks the dopamine transporter (DAT), the key mechanism responsible for the removal of extracellular dopamine (DA), thereby elevating extracellular DA levels in various limbic, striatal, cortical, cerebellar terminal fields and increasing DA signaling and duration of DA response [2]. Neuroimaging studies showed that therapeutic doses of methylphenidate increased DA levels in the striatum and nucleus accumbens (NAc), a mechanism purportedly thought to explain methylphenidate-induced improvement of ADHD symptoms [3,4]. However, in light of the observation that the effects of methylphenidate are similar to those exerted by other drugs affecting the CNS (e.g. addictive drugs such as cocaine or methamphetamine), the abuse potential of methylphenidate has been suggested [5].

The repeated use of stimulants can elicit adverse effects in behavior and induce drug-related behaviors such as sensitization, tolerance or dependence [6-8]. Therefore, the propensity of long-term methylphenidate treatment to induce drug addiction or dependence in individuals with ADHD has also been implied. A number of studies have investigated effects of methylphenidate treatment on subsequent abuse of other addictive drugs [9-13]. Unfortunately, however, only a few studies have sought to determine whether the repeated treatment of methylphenidate will result in the abuse of the drug itself. Furthermore, the long-term effects of repeated methylphenidate use in behavior and gene expression remain far from understood [14]. In particular, there is a dearth of information on the potential neuronal correlates of long-term recreational methylphenidate use or abuse in individuals with ADHD [8,14].

Preclinical studies in animal models have provided an avenue for identifying the potential molecular neuropathobiology of drug-induced neuroadaptations [15]. Moreover, the use of microarray expression profiling has aided us in capturing neuroadaptations involving complex changes in gene expression that may underlie the development of drug addiction [15,16]. The early microarray studies have shown that repeated methylphenidate treatment even at a clinically relevant dose produced changes in gene regulation in cortical and striatal neurons which are similar to those of cocaine and amphetamine, indicating addiction liability of the drug [14]. While identification of these gene sets represents a significant step, it is likely that there are other changes that have not been identified, especially those that result from the behavioral or cognitive processes associated with methylphenidate use, in addition to the direct pharmacological effects of the drug [17-19]. Indeed, by integrating methylphenidate-induced gene expression changes with outcomes in “appropriate” animal models of drug addiction (e.g. conditioned place preference [CPP] tests and drug self-administration), we identified a subset of neuronal development genes, which may also mediate the reinforcing effect of methylphenidate [19]. We suggested significant clinical implications of these findings on the abuse of methylphenidate among “healthy” individuals, as our studies [19] were conducted in Wistar rats, strain used to represent the “normal” heterogeneous population. However, the previous findings could not be generalized to individuals with ADHD who are also abusing methylphenidate, as experiments were not conducted in “appropriate” ADHD animal models.

To address this issue at the preclinical level and to shed some light on the potential molecular consequences of long-term recreational methylphenidate use or abuse in individuals with ADHD, we analyzed the differentially expressed genes (DEGs) in the brain of methylphenidate-pretreated Spontaneously Hypertensive rats (SHRs), which showed CPP to and self-administration of methylphenidate. The SHR, relative to the normotensive Wistar Kyoto rat strain, exhibits good face, construct and predictive validity mimicking the behavioral characteristics observed in ADHD, and is considered as the most widely used and validated animal model for this disorder [20,21]. Abnormalities both in the genetic and neurotransmitter functions, such as those seen in ADHD were also observed in the SHR [22,23]. Moreover, earlier studies have found increased reactivity of the SHR to stimulants, opioids, alcohol and other addictive drugs in comparison with other strains, indicating that the SHR, in general, may be used to investigate the relationship between ADHD and drug addiction [24]. Most studies on the molecular effects of stimulants have focused on gene regulation in DA target areas such as the striatum [25,26]. In this regard, genome-wide transcriptional analyses were performed in the striatum because this brain region controls reward sensitivity, motor function and habit learning [27]. Furthermore, prefrontal cortices (PFC) were also included in these analyses because they include regions involved in processes relevant to drug addiction such as compulsive drug taking, and maintenance of behavioral sensitization [28,29].

## Methods

### Animals and drug treatment

All experiments were performed in accordance with the Principles of Laboratory Animal Care (NIH) and the Animal Care and Use Guidelines of Sahmyook University, Korea. Male SHRs were purchased from Orient Co. Ltd., a branch of Charles River Laboratories (Seoul, Korea) and housed in an environmentally-controlled animal room (temperature [22 ± 2°C] and humidity [55 ± 5%], 12 h/12 h light/dark [6 AM-6 PM] cycle) in groups during drug treatment and conditioned place preference (CPP) experiments, and individually thereafter during self-administration tests. After a 1 week of acclimatization, adolescent (6 weeks old) SHRs were given saline [(1 mg/kg, intraperitoneal (i.p.), cohort 1] or racemic *dl*-methylphenidate (obtained from Hwanin Pharmaceutical Co. Korea), twice daily [at 9 AM and 9 PM], for 7 days (cohort 2) at a dose (5 mg/kg, i.p.) that produced a CPP response to the drug [19,30], and evaluated for subsequent methylphenidate CPP and self-administration. Another group of SHRs (Control) was pretreated with saline and given saline only during all behavioral assays. Methylphenidate pretreatment was started during adolescence, in view of the findings that treatment initiation during adolescence increased risk of polydrug use [31,32], and in light of the observation that methylphenidate pre-exposure during adolescence increases behavioral response of rats to the reinforcing effects of addictive drugs [*for review see*[8]]. Furthermore, since our goal was to “model” recreational use of methylphenidate in individuals with ADHD, SHRs were pretreated with methylphenidate at a dose beyond the clinically-relevant range (0.5-2 mg/kg,i.p.), and administered with methylphenidate intraperitoneally, a method which is twice as potent as oral methylphenidate administration in increasing extracellular DA levels [33]. Of note, while drug treatment was conducted during both light and dark phases of the light/dark cycle, all behavioral tests were conducted during the light portion of the day only (between 9 AM and 5 PM).

### Conditioned place preference (CPP) tests

CPP tests, conducted a day after the final drug or saline administration, were performed following the methods described previously [30,34]. Tests were performed in a two-compartment CPP apparatus described in previous studies [30,34]. Animal movement and behavior were video-recorded and analyzed using the Ethovision (Noldus, Netherlands) system. The CCP tests consisted of three phases: habituation and preconditioning (3 days), conditioning (6 days) and post-conditioning (1 day). After two days of habituation sessions, initial preference of rats for one compartment of the CPP box was measured for 15 minutes. The conditioning phase followed (6 days) during which cohort 1 and 2 rats were injected with methylphenidate (5 mg/kg, i.p.) and confined to their initially non-preferred side for 30 min [30,34], and given saline (1 mg/kg, i.p.) and confined to their preferred side on alternate days. Meanwhile, rats of the control group received saline only during the conditioning days. The post-conditioning phase followed the next day and the time spent by each rat in each compartment of the CPP apparatus was recorded for 15 min. After the post-conditioning phase, the shift in place preference was determined. CPP data were expressed as the difference in time spent in the drug- or saline-paired compartment during the post and preconditioning phases. The results were presented as the means ± SEM and statistical analysis was performed using unpaired *t*-tests. A *P* value of <0.05 was regarded as significant.

### Self-administration tests

The rats that showed robust CPP to methylphenidate were used for further tests (*n* = 7-9 rats per group) [19]. Six rats from the control group were also used in this study. Rats were trained to press a lever for a sucrose pellet reward and when lever-pressing behaviors were stabilized, they were implanted with silastic catheters in the right jugular vein following methods described in our previous study [34]. Following recovery from surgery, rats underwent 6 days of 2-h methylphenidate or saline (control group) self-administration under the FR1 schedule. The methods of the self-administration tests are outlined in our previous study [34]. Each active lever press resulted in an infusion of 0.1 ml saline (0.9% NaCl), or methylphenidate (0.25 mg/0.1 ml infusion) [34] for cohort 1 or 2 groups, respectively. The number of saline or methylphenidate infusions administered by rats over the 6 days of self-administration was recorded. The results are presented as the means ± SEM and two-way ANOVA was used for data comparison. A *P* value of <0.05 was regarded as significant. All statistical analyses (CPP and self-administration tests) were conducted using GraphPad Prism Version 5 software (San Diego, CA, USA).

### Tissue collection and RNA preparation

One day after the final self-administration session, we removed the brains of 3 rats from cohort 2 (i.e., those which showed the most robust self-administration) as well as 3 rats from the control group for microarray analyses. We used this time point to eliminate confounding results induced by the direct effects of the drug [19]. After decapitation, the brains were rapidly removed and placed in ice-cold saline. The striatum (rostral part of the caudate, putamen and the NAc) and the prefrontal cortex were removed and immediately frozen at −70°C. Total RNA was isolated using Trizol reagent (Invitrogen, Carlsbad, CA, USA) according to the manufacturer’s instructions. The RNA was further purified using the RNEasy mini kit (Qiagen Inc.). The total RNA concentration was determined using NanoDrop ND-1000 Spectrometer (NanoDrop Technologies Inc., Montchanin, DE, USA). The RNA integrity was assessed using a BioAnalyzer 2100 (Agilent Technologies, Santa Clara, CA, USA).

### Oligonucleotide microarray analyses

Affymetrix GeneChip microarrays were prepared, hybridized and scanned by the local authorized Affymetrix service provider (DNA Link, Inc., Seoul, South Korea). The RNA was converted to cDNA and transcribed into cRNA in the presence of biotinylated ribonucleotides, according to standard Affymetrix protocols (Affymetrix ‘Expression Analysis Technical Manual’, #701021 Rev. 5). Biotin-labeled aRNA were transcribed in vitro following the Affymetrix GeneChip manufacturer’s protocol. Hybridization was performed using Affymetrix GeneChip Rat Genome 230 2.0 oligonucleotide arrays (Affymetrix, Santa Clara, CA, USA). The hybridized probe array was stained and washed with a GeneChip hybridization, wash and stain kit using the Fluidics Station 450 (Affymetrix). The stained GeneChip probe array was scanned with a GeneChip Scanner 3000 + 7G (Affymetrix). The signal intensity of the gene expression level was calculated using Expression Console Software, Version 1.1 (Affymetrix) based on the MAS 5.0 algorithm. The procedure used to select DEGs was as follows: (i) selection of “present” Affymetrix probe sets for 3 baseline or treated tissue samples; (ii) selection of “up-regulated” and “down-regulated” Affymetrix probe sets with comparison signal sample/control ratios >1.65 and <0.6, respectively, and whose values corresponded to average ratio values at the 2.5% tails of three microarray histograms; and (iii) selection of Affymetrix probe sets with simultaneously significant *P* values (threshold, 0.05) in the Wilcoxon rank test when compared with vehicle-treated samples.

### Gene ontology analysis

The Database for Annotation, Visualization and Integrated Discovery (DAVID) v6.7 functional clustering tool was used to identify over-represented ontologic groups among the gene expression profiles and to group DEGs into functional categories [35]. Gene Ontology Biological Process was selected as the functional annotation category for this analysis.

### Validation of microarray data by qRT-PCR

qRT-PCR was performed to validate microarray data on selected genes. Briefly, 1 microgram of total RNA, obtained from samples used for microarrays, was reverse-transcribed into cDNA using SuperScript TM reverse transcriptase (Invitrogen), and aliquots were stored at −20°C. The gene identification number for the TaqMan probes (Applied Biosystems) used in the qRT-PCR analyses are shown in Table 1. Rat GAPDH (Applied Biosystems) was also amplified to normalize the variations in cDNA quantities from different samples. The qRT-PCR reactions were performed in triplicates, and the qRT-PCR data were represented as Ct values, where Ct is defined as the threshold cycle. The relative differences in gene expressions were quantified using equations from a previously developed mathematical model [36]. The results are presented as the means ± SEM, and statistical analysis was performed using one-way ANOVA followed by Bonferroni’s post-test. A *P* value of <0.05 was regarded as significant.

**Table 1:** Gene identification numbers for Taqman probes used in the qRT-PCR analysis

**Gene symbol**	**Probe set ID**
*Angptl4*	Rn01528817_m1
*Cttnd1*	Rn01486371_m1
*Gapdh*	Rn01775763_g1
*Homer1*	Rn00581785_m1
*Nedd4*	Rn01530544_m1
*Pcdh10*	Rn01294983_m1
*Perp*	Rn01440467_m1
*S100a9*	Rn00585879_m1
*Uqcrc2*	Rn01506370_m1

## Results

### Reinforcing effect of repeated methylphenidate treatment in SHRs

**Figure 1:**
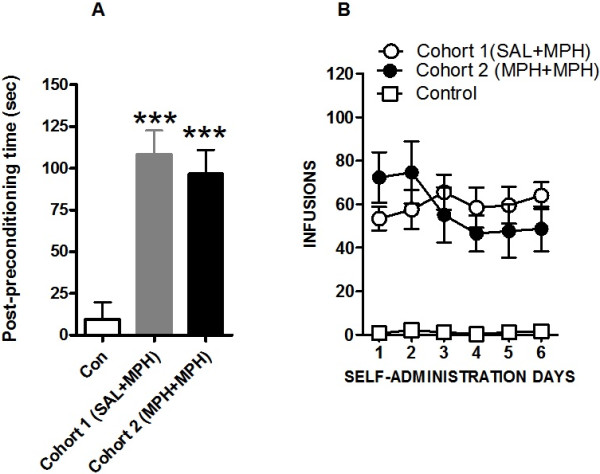
**Reinforcing effects of repeated methylphenidate treatment in spontaneously hypertensive rats (SHRs). (A)** Methylphenidate (MPH) CPP in cohort 1 (saline-pretreated SHR conditioned with methylphenidate, SAL + MPH) and cohort 2 (methylphenidate-pretreated SHR conditioned methylphenidate, MPH + MPH) rats. During the conditioning phase of the CPP test, the rats in both cohorts were administered 5 mg/kg dose of MPH. The control group received saline only. Each bar represents the mean ± S.E.M. of the difference in the time spent in the drug- or saline- (for control group) paired side during the post- and preconditioning phases. ***P < 0.001, significantly different from the saline-treated groups. *n* = 10-12 rats per group. **(B)** MPH self-administration shown by saline-treated (SAL + MPH) and methylphenidate-pretreated (MPH + MPH) SHR. The mean number of methylphenidate infusions obtained by rats over the 6 days of methylphenidate self-administration is shown. Each symbol represents the mean ± S.E.M. *n* = 7-9 animals per group.

Figure 1 shows that methylphenidate-CPP was expressed by methylphenidate-pretreated SHRs conditioned with methylphenidate (cohort 2) [*t* (20) = 4.17, *P* < 0.001] (Figure 1). Saline-pretreated SHRs conditioned with the drug (cohort 1) also showed CPP to methylphenidate ([*t* (18) = 5.58, *P* < 0.001] and the average CPP scores between cohort 1 and 2 rats did not vary significantly [*t* (20) = 0.95, *P* > 0.05]. Out of the 12 animals in cohort 2, we selected 9 rats with the highest CPP scores for further self-administration tests. Seven rats from cohort 1 (i.e., rats with the highest CPP scores) and 7 rats from the control group were also chosen for further self-administration studies. Figure 1B shows the number of methylphenidate infusions obtained by SHRs. Self-administration tests showed reinforcing effects of methylphenidate in cohort 2 [*F* (1,84) = 106.0, *P* < 0.001] as well as in cohort 1 SHRs [*F* (1,72) = 333.4, *P* < 0.001] and two-way ANOVA revealed no significant difference in the rate of responding for methylphenidate infusions between the two groups [*F* (1,84) = 0.13, *P* > 0.05]. In summary, the findings from both CPP and self-administration studies indicate reinforcing effect of methylphenidate in drug-pretreated SHRs.

### Transcriptional responses to the reinforcing effect of repeated methylphenidate treatment in the prefrontal cortex and striatum of the SHR

With regard to the objective of this study, we analyzed prefrontal cortical and striatal gene expression changes in methylphenidate-pretreated SHRs, which showed CPP to and self-administration of methylphenidate. Genome-wide transcriptional profiling showed that 30 transcripts were differentially regulated in the PFC (Table 2), while 306 genes were differentially expressed in the striatum of cohort 2 SHRs vs. controls (see Additional file 1: Tables S1A and 1B for complete list of differentially expressed genes in the striatum). Interestingly, a majority (82%) of the DEGs in the striatum were downregulated genes (Additional file 2: Table S2). The differentially expressed genes (DEGs) in the PFC belong to functional categories such as regulation of apoptosis (*Nfkbia, S100a9*, *Angptl4*), transcription (*Cebpb, Dbp, Per3*) and cell migration (*Abcc9, Ctgf*) (Table 2). The main functional categories overrepresented among the upregulated genes in the striatum include those involved in synaptic transmission (*Grm8, Shank1, Camk2n1, Pja2*), negative regulation of apoptosis (*Sap30bp, Apoe, Nfkbia*), transcription (*Dbp, Klf2, Neurod2*), and others (Additional file 2: Table S2). Categories overrepresented among the downregulated genes are those associated with cell adhesion (*Pcdh10, Ctbbd1, Itgb6*), positive regulation of apoptosis (*Perp, Taf1, Api5*), transcription (*Notch3, Nsbp1, Sik1*), mitochondrion organization (*Prps18c, Letm1, Uqcrc2*), ubiquitin-mediated proteolysis (*Nedd4, Usp27x, Ube2d2*) and others (Additional file 2: Table S2).

**Table 2:** Differentially expressed genes in the prefrontal cortex (PFC) of cohort 2 SHRs relative to controls

**A. Upregulated genes**			
**Probe set ID**	**Gene title**	**Gene symbol**	**Mean fold change**
	**Regulation of apoptosis**		
**1388924_at**	**Angiopoietin-like 4**	***Angptl4***	**1.78**
1389538_at	Nuclear factor of kappa light polypeptide gene enhancer in B-cells inhibitor, alpha	*Nfkbia*	1.66
**1387125_at**	**S100 calcium binding protein A9**	***S100a9***	**4.37**
1373302_at	Alkaline ceramidase 2	*Acer2*	2.27
1391791_at	Alkaline ceramidase 2	*Acer2*	1.87
	**Transcription**		
1387087_at	CCAAT/enhancer binding protein (C/EBP), beta	*Cebpb*	1.78
1387874_at	D site of albumin promoter (albumin D-box) binding protein	*Dbp*	2.18
1378745_at	Period homolog 3 (Drosophila)	*Per3*	1.68
	**Protein complex assembly**		
1389234_at	Von Willebrand factor	*Vwf*	2.17
1367553_x_at	Hemoglobin, beta /// beta globin minor gene /// beta-globin	*Hbb /// LOC100134871 /// LOC689064*	2.82
1370239_at	Hemoglobin alpha, adult chain 2 /// hemoglobin alpha 2 chain	*Hba-a2 /// LOC360504*	1.86
1370240_x_at	Hemoglobin alpha, adult chain 2 /// hemoglobin alpha 2 chain	*Hba-a2 /// LOC360504*	1.86
	**Cell migration**		
1398265_at	ATP-binding cassette, subfamily C (CFTR/MRP), member 9	*Abcc9*	1.81
1367631_at	Connective tissue growth factor	*Ctgf*	1.68
	**Miscellaneous**		
1371237_a_at	Metallothionein 1a /// transthyretin	*Mt1a /// Ttr*	1.69
1371447_at	Placenta-specific 8	*Plac8*	1.80
1384969_at	Collagen, type XXIV, alpha 1	*Col24a1*	1.70
1387658_at	Eukaryotic elongation factor-2 kinase	*Eef2k*	1.69
1379766_at	---	*---*	1.66
1381178_at	---	*---*	2.15
1386145_at	---	*---*	2.23
1389250_at	---	*---*	1.71
**B. Downregulated genes**			
**1370454_at**	**Homer homolog 1 (Drosophila)**	***Homer1***	**0.37**
1370997_at	Homer homolog 1 (Drosophila)	*Homer1*	0.46
1380545_at	ArfGAP with SH3 domain, ankyrin repeat and PH domain 1	*Asap1*	0.58
1393324_at	Junctional adhesion molecule 2	*Jam2*	0.55
1393451_at	Centromere protein N	*Cenpn*	0.54
1395309_at	---	*---*	0.60
1378507_at	---	*---*	0.58
1384410_at	---	*---*	0.59

### qRT-PCR validations of selected differentially expressed genes

**Figure 2:**
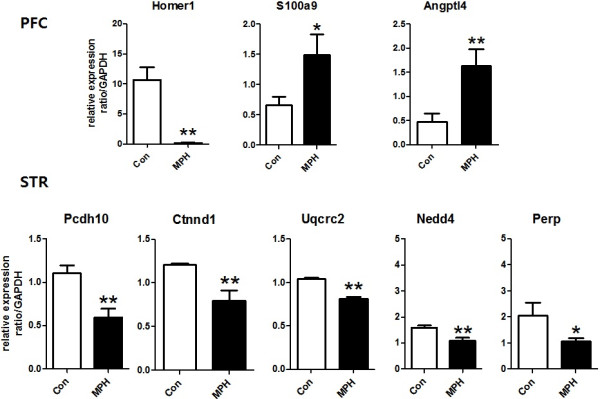
**The confirmed changes in the prefrontal cortical and striatal gene expression in cohort 2 spontaneously hypertensive rats (SHRs).** qRT-PCR validated the expression of *Homer1*, *S100a9* and *Angptl4*, differentially expressed genes (DEGs) in the prefrontal cortex (PFC), as well as *Pcdh10, Ctnnd1, Uqcrc2, Nedd4*, and *Perp*, DEGs in the striatum of cohort 2 SHRs. The data represent the expression ratios with respect to the control group (Con), and the values were normalized to GAPDH. *P < 0.05, **P < 0.01 significantly different from the control groups.

qRT-PCR was used to confirm a subset of differentially expressed genes identified in the microarray studies. The genes selected for qRT-PCR confirmation were those representing the major functional gene families among the downregulated genes. The expression patterns of *Pcdh10* and *Ctnnd1,* genes associated with cell adhesion were validated through qRT-PCR (Figure 2). We also confirmed microarray results with *Perp, Nedd4* and *Uqcrc2*, genes associated with apoptosis, ubiquitin-mediated proteolysis, and mitochondria organization, respectively (Figure 2). Furthermore, we also found a correlation between expression patterns predicted by microarrays and those determined by qRT-PCR analyses for *S100a9, Angptl4 and Homer1*, DEGs in the PFC of cohort 2 SHRs. These findings strengthen the reliability of our microarray results.

## Discussion

To model recreational methylphenidate use in individuals with ADHD, we subjected SHRs to repeated methylphenidate treatment (7 days, twice daily) at a dose (5 mg/kg, i.p.) beyond the clinically-relevant range (0.5-2 mg/kg, i.p.), and tested them for subsequent methylphenidate CPP and drug self-administration. In order to shed light on the potential molecular consequences of long-term recreational methylphenidate use or abuse in individuals with ADHD, we analyzed differentially expressed genes (DEGs) in the PFC and striatum of methylphenidate-pretreated SHRs, which showed methylphenidate reinforcement (i.e. CPP to and self-administration of methylphenidate). The findings from behavioral studies revealed methylphenidate CPP and acquisition of methylphenidate self-administration in methylphenidate-pretreated SHRs. Genome-wide analyses in the PFC and striatum of these rats showed that methylphenidate alters the expression of a number of genes involved in many functional systems. We discuss herein some of these important DEGs whose functions have already been characterized, and hence a role in methylphenidate reinforcement in SHRs can be extrapolated.

Previous studies showed that methylphenidate pretreatment at the 5 mg/kg dosage (i.p.) enhanced psychomotor response to cocaine in other rat strains (e.g. Sprague–Dawley rats and Wistar rats) [10]. Moreover, pretreatment at various dosages of methylphenidate also increased vulnerability to cocaine as well as to other addictive drugs [8,10,11]. We have previously reported enhancement of CPP response to methylphenidate in methylphenidate-pretreated (5 mg/kg, ip., 7 days, twice daily) Wistar rats [19] indicating the occurrence of behavioral sensitization, a phenomenon associated with enhanced behavioral response to repeated treatment of addictive drugs [37]. Drug-induced sensitization has been thought to underlie certain aspects of addiction; in particular, the neuroadaptations that accompany this phenomenon have been suggested to mediate the maintenance and reinstatement of addiction [37]. We observed in this study that methylphenidate pretreatment produced reinforcing effects in SHRs, however, we also found comparable CPP scores and similar rates of methylphenidate self-administration in both saline- and methylphenidate-pretreated rats (cohort 1 and 2 rats, respectively). Investigating the mechanism that underlies differential behavioral responses of strains to the reinforcing effects of repeated methylphenidate treatment is beyond the scope of this study [*for review, however, see*[38]]. Nevertheless, the present and the previous findings [19] appear to simulate the reported similarity in the rates of drug abuse or dependence to psychoactive substances in both ADHD and non-ADHD controls [39].

In general, our microarray studies revealed transcriptional changes associated with pharmacological effects of the drug or consequential to effects of chronic methylphenidate exposure (e.g. genes related with apoptosis, inflammation, etc.), although these analyses also found some DEGs whose expression may have been changed in response to the cognitive or behavioral processes associated with drug use (e.g. genes involved in neuronal and/or synaptic plasticity as well as in neuronal development) [[17,19], *for review see*[18]]. In the PFC, methylphenidate differentially altered the expression of genes involved in apoptosis (e.g. *S100a9, Angptl4, Nfkbia*), transcription (*Cebpb,Per3*), and neuronal plasticity (*Homer1, Jam2, Asap1*). qRT-PCR validated differential expression patterns of *S100a9*, a gene that encodes S100a9, which together with S100a8 exerts broad apoptotic activities [40]. Psychostimulants, including methylphenidate [41], activate the production of superoxides [42], inflammatory cells (astrocytes and microglia), and they in turn have been shown to release numerous pro-inflammatory factors and cytokines [43]. Moreover, psychostimulant-induced increases in synaptic and cytosolic DA levels have been shown to be neurotoxic due to DA-induced production of reactive oxygen species (ROS), reactive nitrogen species, hydrogen peroxide and dopamine quinones [44-46]. Therefore, enhanced expression of apoptosis-related genes in the PFC (as well as in the striatum) of cohort 2 SHRs is consistent with the observed neurotoxic effects of long-term psychostimulant exposure. Additionally, these findings may also reflect other previously described neurotoxic effects of chronic methylphenidate treatment such as decreased antioxidant defenses promoting peripheral oxidative adaptation [47], altered Na^+^, K^+^-ATPase activity in the cerebrum thereby affecting cellular excitability [48], downregulation of activity regulated cytoskeletal gene expression interfering with long-term potentiation and consolidation of long-term memory [49], as well as drug-induced cognitive impairment on spatial reference and working memory tasks [50].

Of note, we observed a larger number of DEGs in the striatum when compared with the PFC in cohort 2 SHRs coinciding with the findings of our previous study [19], and corroborating the previous assumption on the crucial role of the striatum during compulsive drug use or abuse [51]. Interestingly, a majority of the DEGs in the striatum are downregulated genes associated with important cellular functions such as cell adhesion (e.g. *Pcdh10, Ctbbd1, Itgb6*), transcription (e.g. *Notch3, Nsbp1, Sik1*), mitochondrion organization (e.g. *Prps18c, Letm1, Uqcrc2*) and ubiquitin-mediated proteolysis (e.g. *Nedd4, Usp27x, Ube2d2*). The expression patterns of representative genes (e.g. *Pcdh10, Ctnd1, Uqcrc2, Nedd4*) from the different functional categories have been validated by confirmatory qRT-PCR analyses. However, it remains to be established whether changes in expression levels of these genes influenced methylphenidate reinforcement in drug-treated SHRs. Moreover, further studies are warranted to determine the contribution of these altered transcripts in the molecular mechanism of long-term recreational methylphenidate use or abuse in individuals with ADHD.

The decrease in *Pcdh10* gene expression is both a novel and interesting observation considering the role of cell adhesion genes in maintaining neuronal and synaptic connections, and also in neuronal development [52]. *Pcdh10*, a member of the protocadherin gene family of cell adhesion molecules (CAMs), codes for a cadherin-related neuronal receptor involved in the establishment and function of specific cell-cell connections in the brain [53]. Furthermore, mutant analysis has demonstrated that loss of *Pcdh10* can influence different aspects of development and post-natal life [48]. Whereas prior to this study there has been no experimental literature implicating the role of *Pcdh10* in drug abuse, the involvement of this gene in a neurodevelopmental disorder, autism spectrum disorder, has already been reported [54]. Hence, downregulation of *Pcdh10* gene expression observed in this study is an index of altered cell-to-cell communication, which may also signify disturbances in synaptic or neuronal plasticity within the striatal complex.

Altered gene expression of *Uqcrc2* has also been confirmed by qRT-PCR. The *Uqcrc2* gene is a component of the mitochondrial respiratory complex III, which codes for an enzyme involved in the electron transport chain. Furthermore, dysregulation of the expression of this gene has been implicated in some neuropsychiatric disorders such as bipolar disorder [55]. A previous study reported inhibition of mitochondrial respiratory chain complex III in the striatum as well as other brain regions of adult Wistar rats exposed to chronic (28 days) treatment of methylphenidate [56]. Downregulation of mitochondrial respiratory complex III (as well that of other complexes) has been viewed as a compensatory response in order to maintain energy homeostasis through enhancement of other metabolic systems [56]. Alternatively, it has been assumed to result from neurotoxic effects of methylphenidate via the generation of ROS [56]. Further studies are required to examine this point in detail. Nevertheless, the decrease in *Uqcrc2* expression as observed in this study extends the previous findings [56], and indicates that methylphenidate treatment alters brain mitochondrial functions. These findings also suggest disruptions in brain energy metabolism in methylphenidate-treated and reinforced SHRs, although additional studies are required to confirm this.

*Nedd4* gene expression has also been decreased in the striatum of cohort 2 SHR. *Nedd4* belongs to a family of ubiquitin ligases, which has been shown to play key roles in both trafficking and degradation of proteins. In particular, *Nedd4* has been hypothesized to maintain target protein at the plasma membrane by balancing steady state insertion and retrieval of proteins critical to the development and neuronal function in the brain [57]. Although further studies are required, downregulation of *Nedd4* in this study may indicate disruption in the removal of misfolded or unwanted proteins causing their accumulation. The pathological consequences of this altered process in the context of long-term methylphenidate abuse are still unknown, although accumulation of unnecessary proteins resulting from a defective ubiquitin dependent proteolysis has been shown to contribute to aggregation events, a pathogenic mechanism in several neurobehavioral and neurodegenerative disorders [58,59]. Furthermore, in view of the important roles of ubiquitin system in synaptic growth and function, downregulation of *Nedd4* may also affect synaptic growth and plasticity, contributing to altered brain function.

## Conclusion

Altered expression of genes associated with apoptosis, cell adhesion, mitochondria organization, ubiquitin-mediated proteolysis, etc. in the PFC and/or striatum of methylphenidate-treated SHRs which showed methylphenidate CPP and self-administration indicate methylphenidate-induced neurotoxicity, alterations in synaptic and neuronal plasticity, energy metabolism and ubiquitin-dependent protein degradation. In addition, these changes may also reflect cognitive impairment associated with chronic methylphenidate use as demonstrated in preclinical studies [*e.g.*[49,50]]. Future studies should be performed to determine the link between changes in the expression of these genes and influence on methylphenidate reinforcement in SHRs. Moreover, additional studies are warranted to determine the clinical significance of the findings of this study with regard to methylphenidate abuse or addiction in individuals with ADHD. Nevertheless, this study presents new research directions and interesting topics for further investigation. For instance, it would be worthwhile to examine molecular changes that occur in the brains of methylphenidate-pretreated and methylphenidate-reinforced WKY. Providing answers to this query will establish whether the reported gene expression changes are exclusive to SHR and not possibly due to inbreeding. Furthermore, investigating gene expression changes in methylphenidate-pretreated animals which showed less methylphenidate CPP and self-administration will also provide important information not only on the mechanisms of methylphenidate abuse in ADHD individuals, but also on protection against methylphenidate addiction via development of novel targets for the treatment of methylphenidate or psychostimulant addiction. Along these lines, complementary findings can be obtained by comparing gene expression patterns associated with adolescent exposure to methylphenidate and atomoxetine, another widely-used ADHD drug without stimulant-like effects and assumed to be devoid of abuse liability, in view results from a previous study which showed differential effects of these drugs in SHRs [60].

## Competing interests

The authors declare that they have no competing interests.

## Authors’ contributions

ID, JHC and MSN conceived and designed the study. ID, HJK and ARS performed the SA and CPP experiments. ID, MSN and JHC analyzed the microarray data and ID, ARS performed the statistical analysis. JHR, CYS, JHC, BNK and DHH provided insights on interpreting the results. All authors participated in drafting and in writing the final revision of the manuscript. All authors read and approved the final manuscript.

## Supplementary Material

Additional 1: Table S1A Upregulated genes in the striatum. **Table S1B.** Downregulated genes in the striatum. Click here for file

Additional 2: Table S2 Differentially expressed genes in the striatum of cohort 2 SHRs relative to control. Click here for file
